# Effectiveness of Mycophenolate Mofetil Trough Level Monitoring in Children with Relapsing Nephrotic Syndrome

**DOI:** 10.2215/CJN.0000000824

**Published:** 2025-09-16

**Authors:** William Morello, Silvia Bernardi, Giuseppe Puccio, Anita Sofia Bellotti, Evgenia Preka, Mathilde Grapin, Maud Prévot, Marina Charbit, Teresa Nittoli, Maurizio Gallieni, Luciana Ghio, Alberto Edefonti, Olivia Boyer, Giovanni Montini

**Affiliations:** 1 Pediatric Nephrology, Dialysis and Transplant Unit, Fondazione IRCCS Ca' Granda, Ospedale Maggiore Policlinico, Milan, Italy; 2 Pediatric Nephrology, Reference Center for Idiopathic Nephrotic Syndrome in Children and Adults, University Hospital Necker-Enfants Malades, Assistance Publique - Hôpitaux de Paris (APHP), Imagine Institute, INSERM U1163, Paris Cité University, Paris, France; 3 Department of Biomedical and Clinical Sciences, University of Milan, Milan, Italy; 4 Department of Clinical Sciences and Community Health, Dipartimento di Eccellenza 2023-2027, University of Milan, Milan, Italy

**Keywords:** children, idiopathic nephrotic syndrome, mycophenolate mofetil

## Abstract

**Key Points:**

- Therapeutic drug monitoring on the basis of mycophenolic acid (MPA) trough levels is associated with higher effectiveness of mycophenolate mofetil in maintaining remission in children with steroid-dependent nephrotic syndrome/frequently relapsing nephrotic syndrome.
- Dose adjustments on the basis of MPA trough levels personalize treatment, are associated with higher effectiveness, and help minimize potential toxicity.
- Maintaining MPA trough levels above 2.9 *µ*g/ml provides a relapse-free survival rate more than 85%, similar to that of more toxic drugs.

**Background:**

The effectiveness of therapeutic drug monitoring (TDM) of mycophenolic acid (MPA) trough levels in children with steroid-dependent nephrotic syndrome (SDNS)/frequently relapsing nephrotic syndrome (FRNS) treated with mycophenolate mofetil (MMF) has not been adequately assessed.

**Methods:**

We performed an international, retrospective study including children with SDNS/FRNS treated with MMF as the first-line steroid-sparing agent and a follow-up of more than 6 months. Patients were categorized into two groups: TDM, if MPA trough levels were monitored, and no-TDM, if not. In the TDM group, MMF doses were adjusted to maintain MPA trough levels of more than 3 *µ*g/ml, unless toxicity occurred. The primary outcome was relapse-free survival.

**Results:**

A total of 167 patients were observed, 90 in the TDM group and 77 in the no-TDM group. Relapse-free survival over the total follow-up was significantly longer in the TDM group (*P* = 0.001, log-rank test) with an estimated relapse-free survival at 6 months of 73% for the TDM group and 55% for the no-TDM group. After correcting for potential confounders, the association remained statistically significant (*P* < 0.001). TDM patients also received lower doses of prednisone after MMF introduction. In the TDM group, children were more likely to modify their initial dose (90% versus 9%; *P* < 0.001). Although MMF dose was not associated with relapse (median 1186 versus 1298 mg/m^2^; *P* = 0.14), MPA trough levels were significantly higher in children who did not relapse (4.0 versus 2.7 *µ*g/ml, *P* = 0.001). Among children maintaining mean MPA levels more than 2.9 *µ*g/ml, relapse-free survival at 6 months was 86%. Reported side effects were similar in both groups.

**Conclusions:**

Monitoring MPA trough levels was associated with an approximately 20% higher MMF effectiveness in maintaining remission at 6 months in children with SDNS/FRNS. Personalized MMF dosing, adjusted to maintain MPA levels more than 2.9 *µ*g/ml, was both safe and effective. We recommend including MPA trough level monitoring in future studies comparing MMF with other steroid-sparing agents in children with SDNS/FRNS.

## Visual Abstract

**Figure:**
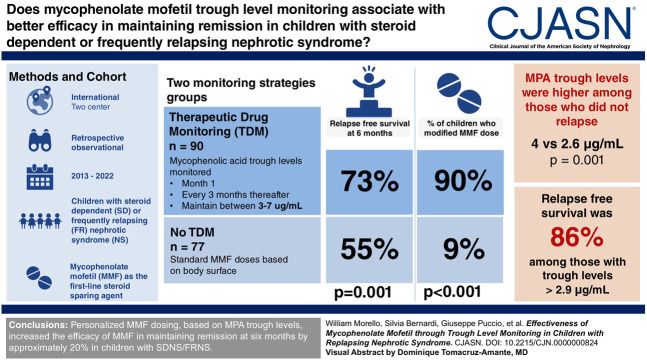

## Introduction

Over 90% of children with idiopathic nephrotic syndrome (INS) are sensitive to steroids. However, most will become frequently relapsing nephrotic syndrome (FRNS) or steroid-dependent nephrotic syndrome (SDNS).^1^ These children often require immunosuppressive steroid-sparing medications to prevent severe multisystemic complications associated with prolonged steroid therapy.^1^

Mycophenolate mofetil (MMF) is a prodrug of mycophenolic acid (MPA), and its safety and efficacy as a steroid-sparing agent in SDNS/FRNS have been extensively documented.^2^ Most guidelines recommend a daily dose of 1200 mg/m^2^ in two divided doses.^3^

Therapeutic drug monitoring (TDM) using MPA area under the curve (MPA-AUC) may improve the effectiveness of MMF in patients affected by SDNS/FRNS.^4^ However, this method requires at least three subsequent measurements^5^, limiting its applicability, particularly in children. TDM on the basis of MPA trough levels requires only one blood sample collected before the next dose, reducing patient discomfort, resource consumption, and costs. Although this method is already used in some centers, its performance has not been adequately assessed.

This study aimed to explore whether TDM on the basis of MPA trough levels is associated with higher effectiveness of MMF in maintaining remission in children with SDNS or FRNS.

## Methods

We performed an international, two-center, retrospective, observational study. The inclusion criteria were (*1*) a clinical diagnosis of SDNS/FRNS according to international guidelines^3^, (*2*) age between 1 and 18 years, (*3*) treatment with MMF as the first-line steroid-sparing agent, initiated between January 2013 and December 2022, (*4*) a follow-up of at least 6 months after the introduction of MMF, and (*5*) signed informed consent obtained from parents or legal guardians. Steroid-resistant patients or children with incomplete data on the primary outcome were excluded. Missing values in predictor variables (covariates) were addressed using complete-case analysis in the multivariate models. Patients were enrolled at the Pediatric Nephrology Units of Ospedale Maggiore Policlinico of Milan (Italy) and Necker-Enfants Malades Hospital in Paris (France) using the data warehouse Dr. Warehouse.^6^ At the time of data collection, MPA trough levels were routinely monitored in most patients in one center and occasionally in the other. Children from both centers were categorized into two groups: the TDM group, if MPA trough levels were monitored, and the no-TDM group, if not. In the TDM group, MPA levels were monitored 1 month after MMF introduction and on a 3-month basis thereafter. MMF doses were adjusted to maintain MPA trough levels between 3 and 7 *µ*g/ml, unless toxicity occurred. Only MPA samples collected between 11 and 13 hours after the last MMF dose were considered valid and included in the analysis; samples obtained outside this window were excluded. The reported median MPA trough level refers to the median of each individual's mean MPA trough level within the first 6 months of treatment, excluding samples collected after relapse.

In the no-TDM group, children were assessed every 3 months. In both groups, at each assessment, relevant clinical data including relapses and adverse events were recorded. Proteinuria was measured by spot urine sampling, and cell blood count and serum albumin levels were assessed. Data were collected from patients' clinical records.

The study was approved by the Ethics Committees of both institutions (code 0032316—March 14, 2024-AIFA-AIFA_STDG-P). Informed consent was obtained from parents or legal guardians, as required.

### Statistical Methods

A time-to-event analysis was performed. The primary end point was the occurrence of the first relapse after MMF introduction in the two study groups (TDM versus no TDM) at 6 months after MMF introduction and through the available follow-up.

Secondary end points were the dose of corticosteroid after MMF introduction in the two study groups (TDM versus no TDM), the association between the two different forms of the disease (SDNS and FRNS) and relapse rate after MMF introduction in the whole population and the two study groups (TDM versus no TDM), the prescribed dose of MMF in the two study groups (TDM versus no TDM), the association between MPA trough levels and the relapse rate (only in the TDM group), and the number of severe adverse events related to MMF in the two study groups (TDM versus no TDM).

The following variables were collected: relevant demographic data (age, sex, and race), INS subgroup (SDNS/FRNS), date of disease onset, number of relapses and cumulative dose of prednisone (PDN)/year per patient before and after the introduction of MMF, date of MMF introduction, TDM for MPA when available, and adverse events.

Categorical variables were summarized as frequencies and percentages. Numerical variables were summarized as medians and interquartile ranges (IQRs). The association between categorical variables was analyzed by the chi-squared test for independence. Differences in distribution of a continuous variable in groups were analyzed using nonparametric methods (Wilcoxon test for independent samples).

For binary responses, simple and multiple logistic regression models were used. Survival analysis was performed using the Kaplan–Meier estimator and log-rank tests. For multiple regression, Cox proportional hazard models were used.

Potential confounders (number of relapses in the 6 months preceding MMF, age at MMF introduction, race, INS subgroup [SDNS versus FRNS], and individual daily PDN dose from disease onset to MMF initiation) were added to multiple regression models according to their unbalanced distribution in the study groups, their potential association with the response, and their potential roles in the biologic and clinical context.

Multiple logistic regression and multiple Cox regression analyses were performed using complete cases only.

All statistical analyses were performed using the open source statistical software R (R Core Team, 2022). R: A language and environment for statistical computing. R Foundation for Statistical Computing, Vienna, Austria. https://www.R-project.org/.

## Results

**Figure 1:**
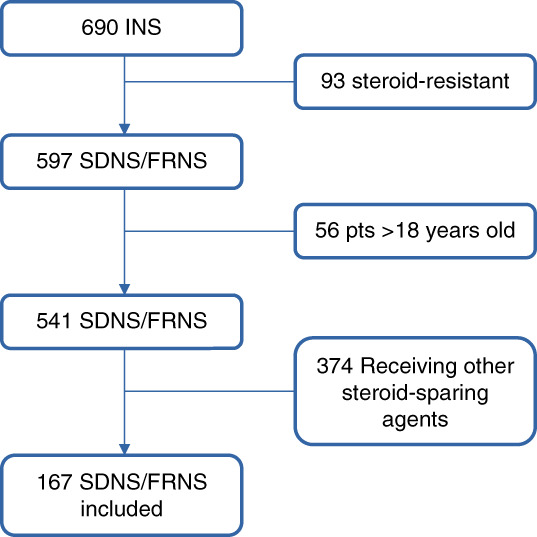
**Study flow chart.** FRNS, frequently relapsing nephrotic syndrome; INS, idiopathic nephrotic syndrome; SDNS, steroid-dependent nephrotic syndrome.

We identified 690 children with INS, of whom 167 were enrolled (Figure 1), including 90 in the TDM group and 77 in the no-TDM group. Ninety-one (55%) patients were male, 99 (59%) were White, and most were affected by SDNS (129; 77%). The median age at onset was 4.2 years (IQR, 2.5–6.8), whereas the median age at MMF introduction was 5.9 years (IQR, 3.5–8.7). In the 6 months preceding MMF, both groups experienced a median of 2 (IQR, 2–3) relapses. Baseline characteristics of the enrolled population are summarized in Table 1.

**Table 1:** Baseline characteristics of enrolled patients

Variable	All (%), *N*=167	TDM (%), *n*=90	No-TDM (%), *n*=77
**Sex**			
Female	76 (45%)	40 (44%)	36 (47%)
Male	91 (55%)	50 (56%)	41 (53%)
**Race**			
Non-White	68 (41%)	33 (37%)	35 (45%)
White	99 (59%)	57 (63%)	42 (55%)
**Type of INS**			
SDNS	129 (77%)	68 (76%)	61 (79%)
FRNS	38 (23%)	22 (24%)	16 (21%)
Age at onset (yr)	4.2 (2.5–6.8)	3.5 (2.4–6.5)	4.5 (2.7–7.1)
Age at MMF introduction (yr)	5.9 (3.5–8.7)	4.9 (3.2–7.8)	6.7 (4.5–9.7)
No. of relapses 6 mo before MMF	2 (2–3)	2 (2–3)	2 (2–3)
Individual daily PDN dose from onset to MMF (mg/m^2^ per day)	20.7 (11.9–29.5)	22.1 (13.3–31.6)	17.5 (11.4–25.4)

FRNS, frequently relapsing nephrotic syndrome; INS, idiopathic nephrotic syndrome; MMF, mycophenolate mofetil; PDN, prednisone; SDNS, steroid-dependent nephrotic syndrome; TDM, therapeutic drug monitoring.

**Figure 2:**
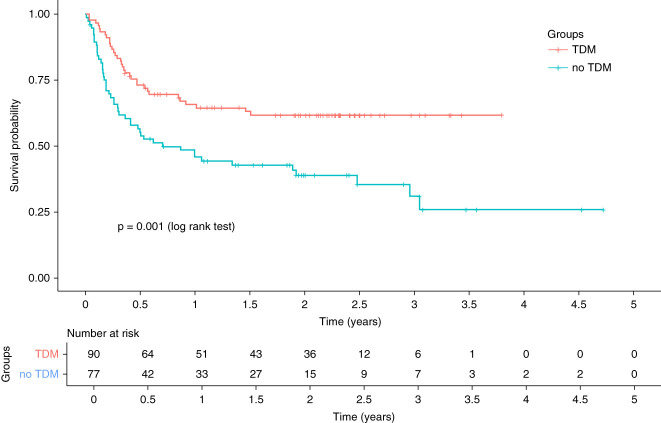
**Kaplan–Meier curve of relapse-free survival in the TDM and no-TDM groups.** Adjustment for multiple comparisons was made using the Holm method. MPA, mycophenolic acid; no-TDM, unmonitored controls; TDM, therapeutic drug monitoring.

The median follow-up was 25.6 months (IQR, 14.0–34.3). Relapse-free survival was significantly longer in the TDM group compared with the no-TDM group (*P* = 0.001, log-rank test; Figure 2). The estimated relapse-free survival at 6 months was 73% for the TDM group and 55% for the no-TDM group. The between-group differences remained statistically significant when comparing children with SDNS and FRNS (Supplemental Figure 1).

After adjusting for potential confounders (number of relapses in the 6 months preceding MMF, age at MMF introduction, race, INS subgroup [SDNS versus FRNS], and individual daily PDN dose from disease onset to MMF initiation), the association between TDM and relapse-free survival remained statistically significant, both in a Cox multiple regression analysis model (*P* < 0.001; Table 2) and in a multiple logistic regression analysis at 6 months (*P* = 0.003; Table 3). Accordingly, after MMF introduction, the TDM group received a significantly lower cumulative PDN dose than the no-TDM group (median 463.86 versus 814.5 mg/m^2^ per year; *P* = 0.02). There were no missing cases for the primary analysis, as per the inclusion criteria. Data for one or two predictors were missing for seven patients (4%).

**Table 2:** Multiple regression analysis by the Cox model

Variables (Reference)	Hazard Ratio	CI	*P* Value
Group (no-TDM)	2.65	1.67 to 4.21	<0.001
No. of relapses in the 6 mo before MMF	1.27	0.98 to 1.65	0.07
Age at MMF introduction	0.99	0.93 to 1.05	0.70
Race (non-White)	3.08	1.91 to 4.94	<0.001^a^
Individual daily PDN dose from onset to MMF	1.03	1.02 to 1.05	<0.001^a^
INS subgroup, SDNS vs FRNS (SDNS)	1.88	0.99 to 3.59	0.05

CI, confidence interval; FRNS, frequently relapsing nephrotic syndrome; INS, idiopathic nephrotic syndrome; MMF, mycophenolate mofetil; PDN, prednisone; SDNS, steroid-dependent nephrotic syndrome; TDM, therapeutic drug monitoring.

^a^ Statistically significant.

**Table 3:** Multiple logistic regression analysis evaluating the risk of relapse within 6 months of initiation of mycophenolate mofetil

Predictors (Reference)	Odds Ratios	CI	*P* Value
Group (no-TDM)	3.13	1.49 to 6.79	0.003^a^
Individual daily PDN dose from disease onset to MMF initiation	1.04	1.01 to 1.07	0.01^a^
No. of relapses in the 6 mo before MMF	1.35	0.88 to 2.13	0.18
Age at MMF introduction	0.99	0.89 to 1.09	0.82
Race (non-White)	4.19	1.98 to 9.29	<0.001^a^
INS subgroup, SDNS vs FRNS (SDNS)	2.60	1.00 to 7.39	0.06

CI, confidence interval; FRNS, frequently relapsing nephrotic syndrome; INS, idiopathic nephrotic syndrome; MMF, mycophenolate mofetil; PDN, prednisone; SDNS, steroid-dependent nephrotic syndrome; TDM, therapeutic drug monitoring.

^a^ Statistically significant.

In the TDM group, patients were more likely to modify the initial dose than those in the no-TDM group (90% versus 9%; *P* < 0.001), with 35 patients (40%) reducing the starting dose on the basis of TDM. The prescribed dose of MMF during the first 6 months after its introduction was significantly higher in the TDM group compared with the no-TDM group (median: 1253 versus 1200 mg/m^2^; *P* < 0.001). In the first 6 months, mean doses ranged from 641 to 2246 mg/m^2^ (IQR, 1146–1432) in the TDM group and from 594 to 1363 mg/m^2^ (IQR, 1000–1200) in the no-TDM group (Table 4).

**Table 4:** Mycophenolate mofetil doses during the first 6 months in the study groups

GROUP	Mean	SD	Median	IQR	Range
TDM	1296	240	1253^a^	1146–1432	641–2246
No-TDM	1098	164	1200^a^	1000–1200	594–1363

IQR, interquartile range; TDM, therapeutic drug monitoring.

a *P* < 0.001 (Wilcoxon test for independent samples).

**Figure 3:**
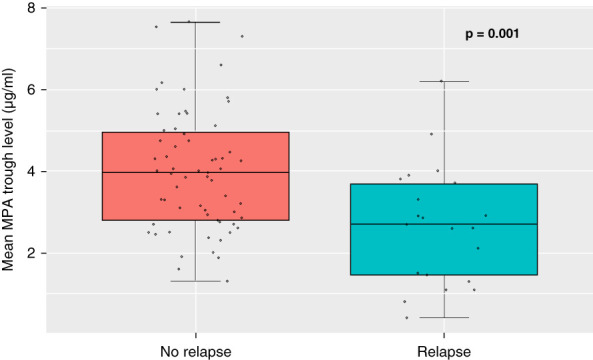
**Distribution of individual mean MPA trough levels during the first 6 months of treatment or before relapse in children with or without relapse in the TDM group.** Boxplots represent the median and IQR of individual means. IQR, interquartile range.

**Figure 4:**
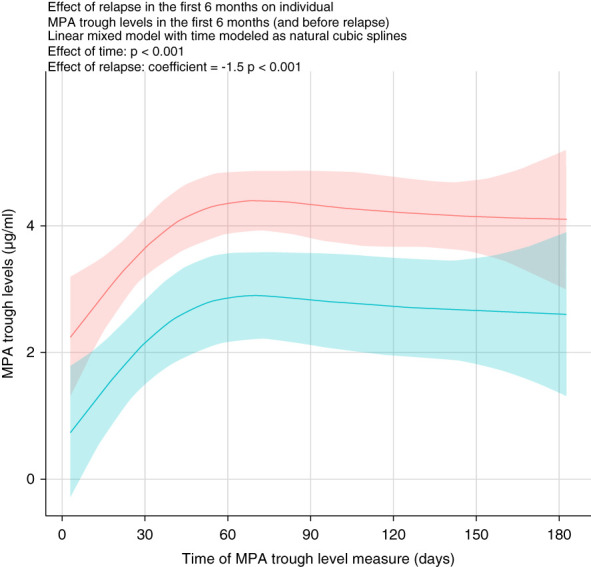
**Association between MPA trough levels and relapse analyzed using a mixed-effects model.** Individual MPA trough levels collected within the first 6 months of treatment were assessed using a mixed-effects model, accounting for repeated measures within patients. Trough levels measured after relapse events were excluded.

In the TDM group, the median MPA trough level was 3.65 *µ*g/ml (IQR, 2.82–4.95). The prescribed MMF dose did not differ significantly between children who experienced a relapse and those who did not within 6 months of treatment (median 1186 versus 1298 mg/m^2^; *P* = 0.14). By contrast, median individual MPA trough levels were significantly higher in children who did not relapse, compared with those who relapsed (median 4.0 versus 2.7 *µ*g/ml, *P* = 0.001; Figure 3). This association remained significant when using the TDM/albumin ratio to correct for serum albumin levels (*P* = 0.005). The results of three additional sensitivity analyses, using the first MPA trough level recorded per patient instead of the average (Supplemental Figure 2), and a mixed-effects model evaluating all TDM levels within the first 6 and 18 months, excluding those obtained after relapse, confirmed the significant association between TDM values and the relapse rate (Figure 4 and Supplemental Figure 3).

**Figure 5:**
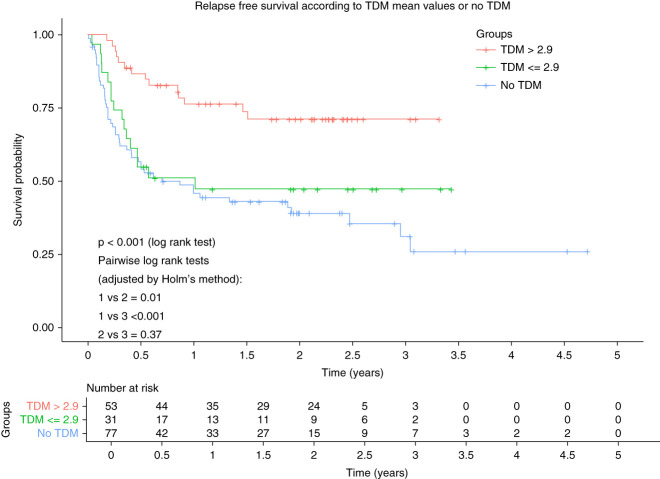
**Kaplan–Meier curve of relapse-free survival of children maintaining a mean MPA trough level more than 2.9 *µ*g/ml compared with those with lower mean through levels and the no-TDM group.** Adjustment for multiple comparisons was made using Holm's method.

In children maintaining mean MPA trough levels more than 2.9 *µ*g/ml, the estimated 6-month relapse-free survival was 86%, whereas in those below this threshold, it was lower and similar to that of patients in the no-TDM group (55% versus 55%, *P* < 0.001; Figure 5).

The incidence of clinically relevant adverse events was similar in both groups (*P* = 0.89; Table 5). In particular, the number of patients experiencing neutropenia and gastrointestinal symptoms was not significantly different between the two study groups. In the TDM group, the incidence of adverse events was not associated with TDM levels (*P* = 0.89).

**Table 5:** Clinically relevant side effects in the study groups

Side Effects	TDM Group (*n*=90)	No-TDM Group (*n*=77)
Gastrointestinal, *No.* (%)	6 (7)	10 (13)
Respiratory tract infections, *No.* (%)	5 (6)	4 (5)
Neutropenia, *No.* (%)	4 (4)	1 (1)
Nonrespiratory viral infections, *No.* (%)	3 (3)	0 (0)
Hyperbilirubinemia, *No.* (%)	0 (0)	1 (1)
Urinary tract infections, *No.* (%)	0 (0)	1 (1)
Total, *No.* (%)	18 (20)^a^	17 (22)^a^
No side effects, *No.* (%)	72 (80)	60 (78)

TDM, therapeutic drug monitoring.

a *P* = 0.89, by chi-squared test.

## Discussion

In this two-center, observational study, we assessed for the first time the effect of TDM performed using trough levels on the effectiveness of MMF in children with SDNS/FRNS. In a large cohort of patients treated with MMF as the first-line steroid-sparing agent, relapse-free survival at 6 months was higher in children who were monitored by trough levels compared with those who were not (73% versus 55%). The difference persisted after correcting for confounders and throughout the long-term follow-up (median 25.6 months).

Our study found that MMF effectiveness is better when dosing is guided by targeted MPA plasma levels. In a *post hoc* analysis performed on 43 children with FRNS enrolled in a German multicenter study, an MPA-AUC more than 50 *µ*g⋅h/ml was associated with a lower relapse rate at 12 months.^4^ Similarly, a retrospective study on 95 children with SDNS/FRNS observed the effectiveness of MPA-AUC in a real-world setting.^5^ However, the need for multiple samplings over several hours limits its applicability, particularly in small children. Although area under the curve remains the pharmacokinetic gold standard, its complexity, cost, and patient burden significantly hinder routine implementation in pediatric practice, even in high-resource settings. Even in the aforementioned study,^5^ only one third of patients had more than one TDM measurement, whereas in our study, we were able to assess TDM every 3 months. As a result, MPA-AUC is not routinely performed in children with SDNS/FRNS or incorporated into clinical trials.^7,8^ Although a limited sampling strategy has been proposed, it still requires at least three samples collected over a minimum of 2 hours.^9^ By contrast, TDM on the basis of trough levels requires only a single sample collected just before the next dose, offering a practical and less invasive alternative, particularly relevant for younger children, where repeated blood draws pose both logistic and ethical challenges.

Previous studies on MPA trough levels in children with INS have been limited by small sample sizes, heterogeneous populations, and the absence of unmonitored control groups.^10^ By including a large cohort with unmonitored controls, our study offers more robust and clinically relevant evidence on the utility of TDM by means of trough levels.

According to our data, this simplified outpatient TDM method may provide a benefit comparable with that of MPA-AUC in improving the effectiveness of MMF in children with SDNS/FRNS,^4^ although additional studies are needed to confirm this finding. The ease of implementation, along with the reduction of discomfort and costs, makes TDM by trough levels a widely applicable approach.

In our study cohort, adjusting the MMF dose on the basis of TDM trough levels resulted in higher median prescribed doses and greater maximum doses compared with the unmonitored group. The maximum dose in the TDM group reached 2246 mg/m^2^ per day, yet this was not associated with increased toxicity compared with the no-TDM group. Moreover, MPA trough levels were not linked to the incidence of adverse events. However, in the TDM group, the median dose did not predict the occurrence of a first relapse, whereas mean trough levels were significantly associated with relapse-free survival. This is likely due to the fact that 40% of children in the TDM group lowered their initial dose. Therefore, MMF dosage in the TDM group was personalized for each patient rather than simply increased, allowing for dose reduction in a significant proportion of patients and minimizing potential toxicity. In children who maintained trough levels of more than 2.9 *µ*g/ml, the relapse-free survival approached 90%, comparable with that achieved with more aggressive steroid-sparing agents.^11^ Interestingly, relapse-free survival in children with subtherapeutic MPA trough levels (≤2.9 *µ*g/ml) was similar to that of the no-TDM group, likely reflecting suboptimal MPA exposure in unmonitored patients, because many children in the TDM group required dose adjustments to achieve target levels.

To further support the robustness of our findings, we performed two sensitivity analyses, both confirming a significant association between MPA exposure and relapse-free survival. These findings suggest that personalizing the MMF dose to target MPA trough levels above 2.9 *µ*g/ml optimizes its effectiveness in children with SDNS/FRNS more effectively than simply administering a fixed higher dose.

Of note, the MPA trough level threshold associated with better outcomes in our study closely matches that identified in adult patients with SLE or ANCA-associated small-vessel vasculitis, where MPA trough levels of more than 3 *µ*g/ml were associated with remission.^12^

Race was significantly associated with relapse, a finding consistent with existing literature on racial disparities in disease course and treatment response in children with INS.^13^ Although this was not the primary focus of our analysis, we accounted for race in all multivariate models, and the association between TDM and MMF effectiveness remained significant. This suggests that the benefit of MPA trough level monitoring may be observed across racial groups.

This study has some limitations. First, its retrospective design may have introduced unknown biases. Patients were treated with MMF as the first steroid-sparing agent on the basis of clinician preference and institutional practice; therefore, selection bias cannot be excluded. Although the no-TDM group had longer disease duration, both groups had similar relapse rates and met the same SDNS/FRNS criteria. The TDM group had higher baseline PDN doses, possibly reflecting greater disease severity. Our multivariate analyses adjusted for these factors, but we acknowledge that some residual confounding may remain. In addition, the two participating centers may have had slightly different management strategies, introducing potential variability. However, conducting the study in two tertiary care centers that uniformly treat patients according to the most recent guidelines should have minimized potential confounders. Finally, despite a median follow-up of 25.6 months, long-term adverse effects associated with higher cumulative MMF exposure were not systematically assessed and may be underrepresented in our analysis.

In conclusion, TDM by trough levels was associated with approximately 20% higher effectiveness of MMF in maintaining remission at 6 months in children with SDNS/FRNS. Using personalized MMF doses to maintain MPA levels above 2.9 *µ*g/ml is both safe and effective, even when exceeding 1200 mg/m^2^ per day. Our results support shifting from fixed standard MMF doses to regular MPA trough level monitoring with dosage adjustments to enhance steroid-sparing effectiveness in children with SDNS/FRNS. We recommend that future studies comparing the effectiveness of MMF with that of other drugs in SDNS/FRNS include MPA trough levels or other TDM methods.

## Supplementary Material

SUPPLEMENTARY MATERIAL

## Data Availability

All original data, including deidentified patient-level data or individual laboratory data measurements, are included in the manuscript and/or supplemental material.

## Supplemental Material

Supplemental Figure 1. Kaplan–Meier curve of relapse-free survival in the TDM and no-TDM groups, in children with SDNS or FRNS. Adjustment for multiple comparisons was made using the Holm method.

Supplemental Figure 2. Association between the first recorded MPA trough level and relapse at 6 months.

Supplemental Figure 3. Association between MPA trough levels and relapse analyzed using a mixed-effects model. Individual MPA trough levels collected within the first 18 months of treatment were assessed using a mixed-effects model, accounting for repeated measures within patients. Trough levels measured after relapse events were excluded.
