# Supplementary material for: Effectiveness of Mycophenolate Mofetil Trough Level Monitoring in Children with Relapsing Nephrotic Syndrome
Source: Clin J Am Soc Nephrol. 2025 Sep 16;20(12):1744–52. doi: 10.2215/CJN.0000000824 (PMC12708389; doi:10.2215/CJN.0000000824)
Supplement: SUPPLEMENTARY MATERIAL [file cjasn-20-1744-s001.pdf]

ASN American Society of Nephrology | JASN Journal of the American Society of Nephrology | KIDNEY360 Accessing Our World From Every Angle | CJASN Clinical Journal of the American Society of Nephrology

ASN Journal Disclosure Form

As per ASN journal policy, I have disclosed any financial relationships or commitments I have held in the past 36 months as included below. I have listed my Current Employer below to indicate there is a relationship requiring disclosure. If no relationship exists, my Current Employer is not listed.

A. Bellotti has nothing to disclose.

I understand that the information above will be published within the journal article, if accepted, and that failure to comply and/or to accurately and completely report the potential financial conflicts of interest could lead to the following: 1) Prior to publication, article rejection, or 2) Post-publication, sanctions ranging from, but not limited to, issuing a correction, reporting the inaccurate information to the authors' institution, banning authors from submitting work to ASN journals for varying lengths of time, and/or retraction of the published work.

Name: Anita Sofia Bellotti
Manuscript ID: CJASN-2025-000616R1
Manuscript Title: EFFECTIVENESS OF MYCOPHENOLATE MOFETIL TROUGH LEVEL MONITORING IN CHILDREN WITH RELAPSING NEPHROTIC SYNDROME
Date of Completion: July 15, 2025
Disclosure Updated Date: July 15, 2025

ASN American Society of Nephrology | JASN Journal of the American Society of Nephrology | KIDNEY360 Accessing Our World From Every Angle | CJASN Clinical Journal of the American Society of Nephrology

ASN Journal Disclosure Form

As per ASN journal policy, I have disclosed any financial relationships or commitments I have held in the past 36 months as included below. I have listed my Current Employer below to indicate there is a relationship requiring disclosure. If no relationship exists, my Current Employer is not listed.

S. Bernardi has nothing to disclose.

I understand that the information above will be published within the journal article, if accepted, and that failure to comply and/or to accurately and completely report the potential financial conflicts of interest could lead to the following: 1) Prior to publication, article rejection, or 2) Post-publication, sanctions ranging from, but not limited to, issuing a correction, reporting the inaccurate information to the authors' institution, banning authors from submitting work to ASN journals for varying lengths of time, and/or retraction of the published work.

Name: Silvia Bernardi
Manuscript ID: CJASN-2025-000616
Manuscript Title: EFFECTIVENESS OF MYCOPHENOLATE MOFETIL TROUGH LEVEL MONITORING IN CHILDREN WITH RELAPSING NEPHROTIC SYNDROME
Date of Completion: June 27, 2025
Disclosure Updated Date: June 27, 2025

ASN
American Society of Nephrology
JASN
Journal of the American Society of Nephrology
KIDNEY360
Accessing Our World From Every Angle
CJASN
Clinical Journal of the American Society of Nephrology

ASN Journal Disclosure Form

As per ASN journal policy, I have disclosed any financial relationships or commitments I have held in the past 36 months as included below. I have listed my Current Employer below to indicate there is a relationship requiring disclosure. If no relationship exists, my Current Employer is not listed.

O. Boyer reports the following:
Employer: Hôpital Necker - Enfants Malades; APHP.Centre; Institut Imagine; Université Paris Cité; Consultancy: Biocodex; Advisory or Leadership Role: Advicenne; Alexion; Alnylam; Biocodex; CSL/Vifor; Novartis; Purespring; Samsung; Santhera; Sobi; Takeda; and Speakers Bureau: Advicenne; Alexion; Alnylam; Biocodex; CSL/Vifor; Novartis; Purespring; Samsung; Santhera; Sobi; Takeda.

I understand that the information above will be published within the journal article, if accepted, and that failure to comply and/or to accurately and completely report the potential financial conflicts of interest could lead to the following: 1) Prior to publication, article rejection, or 2) Post-publication, sanctions ranging from, but not limited to, issuing a correction, reporting the inaccurate information to the authors' institution, banning authors from submitting work to ASN journals for varying lengths of time, and/or retraction of the published work.

Name: Olivia Boyer
Manuscript ID: CJASN-2025-000616
Manuscript Title: EFFECTIVENESS OF MYCOPHENOLATE MOFETIL TROUGH LEVEL MONITORING IN CHILDREN WITH RELAPSING NEPHROTIC SYNDROME
Date of Completion: June 23, 2025
Disclosure Updated Date: June 23, 2025

ASN American Society of Nephrology | JASN Journal of the American Society of Nephrology | KIDNEY360 Accessing Our World From Every Angle | CJASN Clinical Journal of the American Society of Nephrology

ASN Journal Disclosure Form

As per ASN journal policy, I have disclosed any financial relationships or commitments I have held in the past 36 months as included below. I have listed my Current Employer below to indicate there is a relationship requiring disclosure. If no relationship exists, my Current Employer is not listed.

M. Charbit has nothing to disclose.

I understand that the information above will be published within the journal article, if accepted, and that failure to comply and/or to accurately and completely report the potential financial conflicts of interest could lead to the following: 1) Prior to publication, article rejection, or 2) Post-publication, sanctions ranging from, but not limited to, issuing a correction, reporting the inaccurate information to the authors' institution, banning authors from submitting work to ASN journals for varying lengths of time, and/or retraction of the published work.

Name: Marina Charbit
Manuscript ID: CJASN-2025-000616R1
Manuscript Title: EFFECTIVENESS OF MYCOPHENOLATE MOFETIL TROUGH LEVEL MONITORING IN CHILDREN WITH RELAPSING NEPHROTIC SYNDROME
Date of Completion: September 5, 2025
Disclosure Updated Date: September 5, 2025

ASN American Society of Nephrology JASN Journal of the American Society of Nephrology KIDNEY360 Accessing Our World From Every Angle CJASN Clinical Journal of the American Society of Nephrology

ASN Journal Disclosure Form

As per ASN journal policy, I have disclosed any financial relationships or commitments I have held in the past 36 months as included below. I have listed my Current Employer below to indicate there is a relationship requiring disclosure. If no relationship exists, my Current Employer is not listed.

A. Edefonti reports the following:
Employer: IRCCS Fondazione Ca' Granda - Ospedale Maggiore Policlinico

I understand that the information above will be published within the journal article, if accepted, and that failure to comply and/or to accurately and completely report the potential financial conflicts of interest could lead to the following: 1) Prior to publication, article rejection, or 2) Post-publication, sanctions ranging from, but not limited to, issuing a correction, reporting the inaccurate information to the authors' institution, banning authors from submitting work to ASN journals for varying lengths of time, and/or retraction of the published work.

Name: Alberto Edefonti
Manuscript ID: CJASN-2025-000616
Manuscript Title: "EFFECTIVENESS OF MYCOPHENOLATE MOFETIL TROUGH LEVEL MONITORING IN CHILDREN WITH RELAPSING NEPHROTIC SYNDROME
Date of Completion: June 24, 2025
Disclosure Updated Date: June 24, 2025

ASN American Society of Nephrology JASN Journal of the American Society of Nephrology KIDNEY360 Accessing Our World From Every Angle CJASN Clinical Journal of the American Society of Nephrology

ASN Journal Disclosure Form

As per ASN journal policy, I have disclosed any financial relationships or commitments I have held in the past 36 months as included below. I have listed my Current Employer below to indicate there is a relationship requiring disclosure. If no relationship exists, my Current Employer is not listed.

M. Gallieni reports the following:
Employer: University of Milano; Consultancy: Consultancy agreements with CSL Vifor (advisory board for CKD-associated pruritus), BD (CEC committee for clinical studies in the vascular access devices area).; Ownership Interest: Partner (5%) in a University of Milan simulation training start-up company: Huvant (https://huvant.com/).; Honoraria: Honoraria as a speaker in meetings, from CME providers, at the national (Italian) and international levels. Sponsors of such meetings in the past 3 years have been: Vantive, BD, Sanofi, and Vifor Pharma; Advisory or Leadership Role: Journal of Vascular Access (SAGE Publishing). Editor in Chief;; and Other Interests or Relationships: Project for People (Italian NGO: www.projectforpeople.org/) : Member of the board of directors.

I understand that the information above will be published within the journal article, if accepted, and that failure to comply and/or to accurately and completely report the potential financial conflicts of interest could lead to the following: 1) Prior to publication, article rejection, or 2) Post-publication, sanctions ranging from, but not limited to, issuing a correction, reporting the inaccurate information to the authors' institution, banning authors from submitting work to ASN journals for varying lengths of time, and/or retraction of the published work.

Name: Maurizio Gallieni
Manuscript ID: CJASN-2025-000616R1
Manuscript Title: EFFECTIVENESS OF MYCOPHENOLATE MOFETIL TROUGH LEVEL MONITORING IN CHILDREN WITH RELAPSING NEPHROTIC SYNDROME
Date of Completion: July 14, 2025
Disclosure Updated Date: June 23, 2025

ASN American Society of Nephrology
CJASN Kidney360 JASN

ASN Journal Disclosure Form

Date: 7/22/2025

Author: Luciana Ghio

Manuscript ID: CJASN-2025-000616R1

Manuscript Title: EFFECTIVENESS OF MYCOPHENOLATE MOFETIL TROUGH LEVEL MONITORING IN CHILDREN WITH RELAPSING NEPHROTIC SYNDROME

Disclosure Statements

Dr. Ghio had nothing to disclose

ASN American Society of Nephrology · JASN Journal of the American Society of Nephrology · KIDNEY360 Accessing Our World From Every Angle · CJASN Clinical Journal of the American Society of Nephrology

ASN Journal Disclosure Form

As per ASN journal policy, I have disclosed any financial relationships or commitments I have held in the past 36 months as included below. I have listed my Current Employer below to indicate there is a relationship requiring disclosure. If no relationship exists, my Current Employer is not listed.

M. Grapin has nothing to disclose.

I understand that the information above will be published within the journal article, if accepted, and that failure to comply and/or to accurately and completely report the potential financial conflicts of interest could lead to the following: 1) Prior to publication, article rejection, or 2) Post-publication, sanctions ranging from, but not limited to, issuing a correction, reporting the inaccurate information to the authors' institution, banning authors from submitting work to ASN journals for varying lengths of time, and/or retraction of the published work.

Name: Mathilde Grapin
Manuscript ID: CJASN-2025-000616
Manuscript Title: EFFECTIVENESS OF MYCOPHENOLATE MOFETIL TROUGH LEVEL MONITORING IN CHILDREN WITH RELAPSING NEPHROTIC SYNDROME
Date of Completion: June 30, 2025
Disclosure Updated Date: June 30, 2025

ASN
American Society of Nephrology
JASN
Journal of the American Society of Nephrology
KIDNEY360
Accessing Our World From Every Angle
CJASN
Clinical Journal of the American Society of Nephrology

ASN Journal Disclosure Form

As per ASN journal policy, I have disclosed any financial relationships or commitments I have held in the past 36 months as included below. I have listed my Current Employer below to indicate there is a relationship requiring disclosure. If no relationship exists, my Current Employer is not listed.

G. Montini reports the following:
Consultancy: Bayern; Alnyalam; Kiowa Kyrin, Chiesi Farmaceutici, Sandoz; and Advisory or Leadership Role: Bayern, Alylam.

I understand that the information above will be published within the journal article, if accepted, and that failure to comply and/or to accurately and completely report the potential financial conflicts of interest could lead to the following: 1) Prior to publication, article rejection, or 2) Post-publication, sanctions ranging from, but not limited to, issuing a correction, reporting the inaccurate information to the authors' institution, banning authors from submitting work to ASN journals for varying lengths of time, and/or retraction of the published work.

Name: Giovanni Montini
Manuscript ID: CJASN-2025-000616R1
Manuscript Title: EFFECTIVENESS OF MYCOPHENOLATE MOFETIL TROUGH LEVEL MONITORING IN CHILDREN WITH RELAPSING NEPHROTIC SYNDROME
Date of Completion: July 17, 2025
Disclosure Updated Date: June 23, 2025

ASN American Society of Nephrology · JASN Journal of the American Society of Nephrology · KIDNEY360 Accessing Our World From Every Angle · CJASN Clinical Journal of the American Society of Nephrology

ASN Journal Disclosure Form

As per ASN journal policy, I have disclosed any financial relationships or commitments I have held in the past 36 months as included below. I have listed my Current Employer below to indicate there is a relationship requiring disclosure. If no relationship exists, my Current Employer is not listed.

W. Morello reports the following:
Employer: Ospedale Maggiore Policlinico Milano

I understand that the information above will be published within the journal article, if accepted, and that failure to comply and/or to accurately and completely report the potential financial conflicts of interest could lead to the following: 1) Prior to publication, article rejection, or 2) Post-publication, sanctions ranging from, but not limited to, issuing a correction, reporting the inaccurate information to the authors' institution, banning authors from submitting work to ASN journals for varying lengths of time, and/or retraction of the published work.

Name: William Morello
Manuscript ID: CJASN-2025-000616
Manuscript Title: EFFECTIVENESS OF MYCOPHENOLATE MOFETIL TROUGH LEVEL MONITORING IN CHILDREN WITH RELAPSING NEPHROTIC SYNDROME
Date of Completion: June 29, 2025
Disclosure Updated Date: June 29, 2025

ASN American Society of Nephrology
JASN Journal of the American Society of Nephrology
KIDNEY360 Accessing Our World From Every Angle
CJASN Clinical Journal of the American Society of Nephrology

ASN Journal Disclosure Form

As per ASN journal policy, I have disclosed any financial relationships or commitments I have held in the past 36 months as included below. I have listed my Current Employer below to indicate there is a relationship requiring disclosure. If no relationship exists, my Current Employer is not listed.

T. Nittoli has nothing to disclose.

I understand that the information above will be published within the journal article, if accepted, and that failure to comply and/or to accurately and completely report the potential financial conflicts of interest could lead to the following: 1) Prior to publication, article rejection, or 2) Post-publication, sanctions ranging from, but not limited to, issuing a correction, reporting the inaccurate information to the authors' institution, banning authors from submitting work to ASN journals for varying lengths of time, and/or retraction of the published work.

Name: Teresa Nittoli
Manuscript ID: CJASN-2025-000616R1)
Manuscript Title: EFFECTIVENESS OF MYCOPHENOLATE MOFETIL TROUGH LEVEL MONITORING IN CHILDREN WITH RELAPSING NEPHROTIC SYNDROME
Date of Completion: July 15, 2025
Disclosure Updated Date: July 15, 2025

ASN Journal Disclosure Form

As per ASN journal policy, I have disclosed any financial relationships or commitments I have held in the past 36 months as included below. I have listed my Current Employer below to indicate there is a relationship requiring disclosure. If no relationship exists, my Current Employer is not listed.

E. Preka reports the following:
Employer: Université de Paris Cité; INSERM U970, PARCC; Paris Translational Research Centre for Organ Transplantation; Paris, France; and Advisory or Leadership Role: IPTA Education Board Council member.

I understand that the information above will be published within the journal article, if accepted, and that failure to comply and/or to accurately and completely report the potential financial conflicts of interest could lead to the following: 1) Prior to publication, article rejection, or 2) Post-publication, sanctions ranging from, but not limited to, issuing a correction, reporting the inaccurate information to the authors' institution, banning authors from submitting work to ASN journals for varying lengths of time, and/or retraction of the published work.

Name: Evgenia Preka
Manuscript ID: CJASN-2025-000616R2
Manuscript Title: Effectiveness of Mycophenolate Mofetil Trough Level Monitoring in Children with Relapsing Nephrotic Syndrome
Date of Completion: August 10, 2025
Disclosure Updated Date: June 23, 2025

ASN
American Society of Nephrology
JASN
Journal of the American Society of Nephrology
KIDNEY360
Accessing Our World From Every Angle
CJASN
Clinical Journal of the American Society of Nephrology

ASN Journal Disclosure Form

As per ASN journal policy, I have disclosed any financial relationships or commitments I have held in the past 36 months as included below. I have listed my Current Employer below to indicate there is a relationship requiring disclosure. If no relationship exists, my Current Employer is not listed.

M. Prevot has nothing to disclose.

I understand that the information above will be published within the journal article, if accepted, and that failure to comply and/or to accurately and completely report the potential financial conflicts of interest could lead to the following: 1) Prior to publication, article rejection, or 2) Post-publication, sanctions ranging from, but not limited to, issuing a correction, reporting the inaccurate information to the authors' institution, banning authors from submitting work to ASN journals for varying lengths of time, and/or retraction of the published work.

Name: Maud Prevot
Manuscript ID: CJASN-2025-000616R1
Manuscript Title: EFFECTIVENESS OF MYCOPHENOLATE MOFETIL TROUGH LEVEL MONITORING IN CHILDREN WITH RELAPSING NEPHROTIC SYNDROME
Date of Completion: July 29, 2025
Disclosure Updated Date: July 29, 2025

ASN American Society of Nephrology | JASN Journal of the American Society of Nephrology | KIDNEY360 Accessing Our World From Every Angle | CJASN Clinical Journal of the American Society of Nephrology

ASN Journal Disclosure Form

As per ASN journal policy, I have disclosed any financial relationships or commitments I have held in the past 36 months as included below. I have listed my Current Employer below to indicate there is a relationship requiring disclosure. If no relationship exists, my Current Employer is not listed.

G. Puccio reports the following:
Employer: AOUP "Paolo Giaccone" Palermo (Retired); Honoraria: Associazione per il Bambino Nefropatico ABN-Onlus; 20122 Milano; via Commenda 16, Padiglione Litta, lo piano; CF:80136970151; and Advisory or Leadership Role: EMBJ; EuroMediterranean Biomedical Journal.

I understand that the information above will be published within the journal article, if accepted, and that failure to comply and/or to accurately and completely report the potential financial conflicts of interest could lead to the following: 1) Prior to publication, article rejection, or 2) Post-publication, sanctions ranging from, but not limited to, issuing a correction, reporting the inaccurate information to the authors' institution, banning authors from submitting work to ASN journals for varying lengths of time, and/or retraction of the published work.

Name: Giuseppe Puccio
Manuscript ID: CJASN-2025-000616R1
Manuscript Title: EFFECTIVENESS OF MYCOPHENOLATE MOFETIL TROUGH LEVEL MONITORING IN CHILDREN WITH RELAPSING NEPHROTIC SYNDROME,
Date of Completion: July 3, 2025
Disclosure Updated Date: July 3, 2025
