# Supplementary material for: Effectiveness of Mycophenolate Mofetil Trough Level Monitoring in Children with Relapsing Nephrotic Syndrome
Source: Clin J Am Soc Nephrol. 2025 Sep 16;20(12):1744–52. doi: 10.2215/CJN.0000000824 (PMC12708389; doi:10.2215/CJN.0000000824)
Supplement: SUPPLEMENTARY MATERIAL [file cjasn-20-1744-s002.pdf]

Supplemental material for "Effectiveness of Mycophenolate Mofetil Trough Level Monitoring in Children with Relapsing Nephrotic Syndrome"

TABLE OF CONTENTS

Page 2 - Supplementary Figure S1. Kaplan-Meier curve of relapse-free survival in the TDM and No-TDM groups, in children with steroid-dependent (SDNS) or frequent relapsing nephrotic syndrome (FRNS). Adjustment for multiple comparisons was made using Holm's method. TDM, therapeutic drug monitoring. No-TDM, unmonitored controls. MPA, mycophenolic acid.

Page 3 - Supplementary Figure S2. Association between the first recorded mycophenolic acid (MPA) trough level and relapse at 6 months.

Page 4 - Supplementary Figure S3. Association between mycophenolic acid (MPA) trough levels and relapse analyzed using a mixed-effects model. Individual MPA trough levels collected within the first 18 months of treatment were assessed using a mixed-effects model, accounting for repeated measures within patients. Trough levels measured after relapse events were excluded.

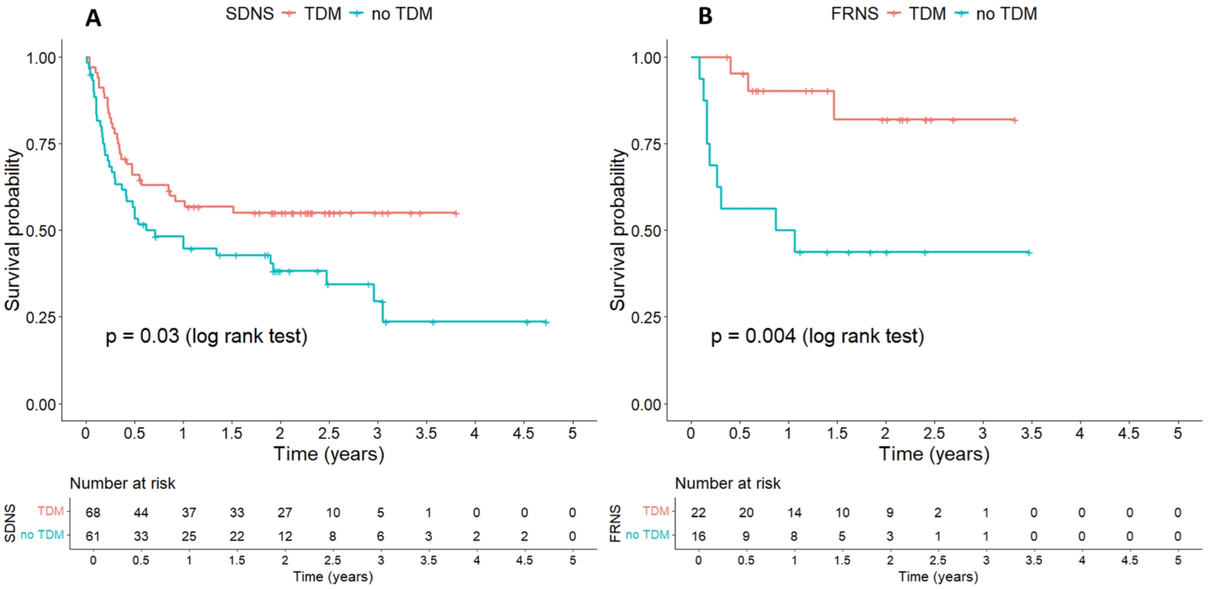

Supplementary Figure S1 – Kaplan-Meier curve of relapse-free survival in the TDM and No-TDM groups, in children with steroid-dependent (SDNS) or frequent relapsing nephrotic syndrome (FRNS). Adjustment for multiple comparisons was made using Holm's method. TDM, therapeutic drug monitoring. No-TDM, unmonitored controls. MPA, mycophenolic acid.

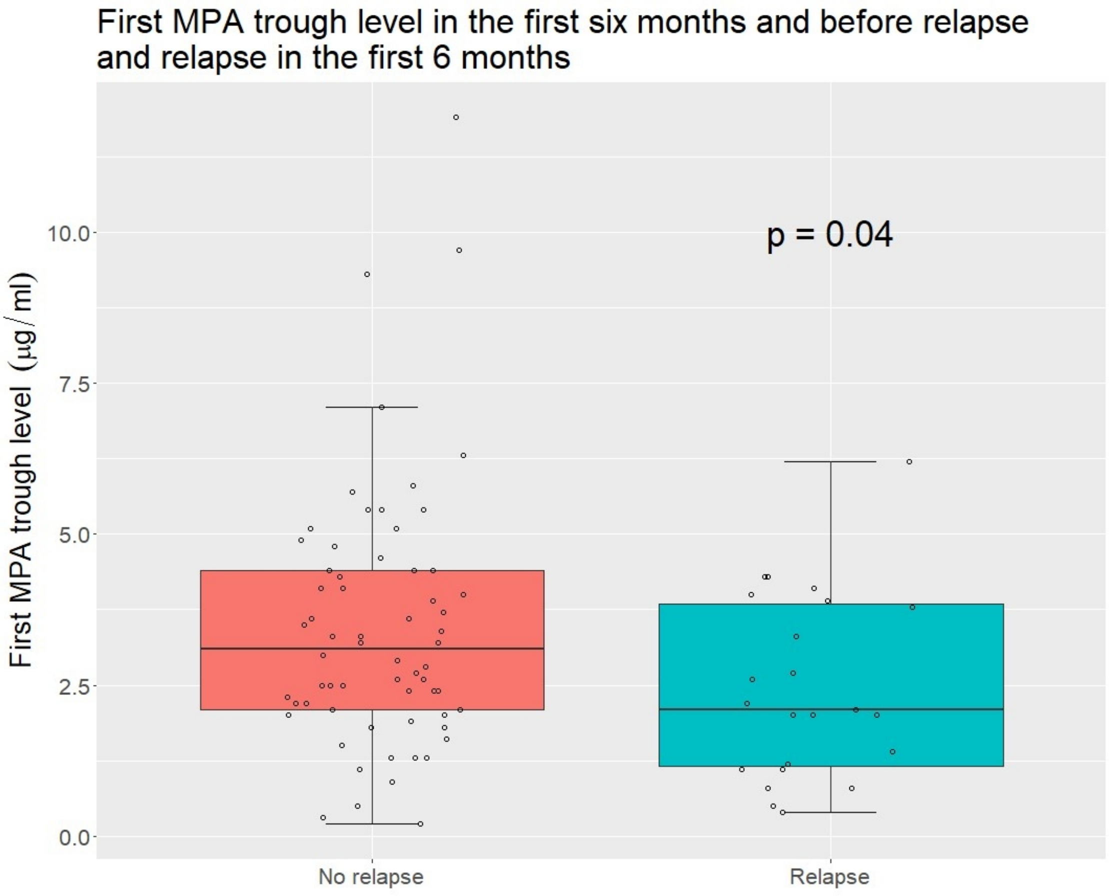

Supplementary Figure S2 - Association between the first recorded mycophenolic acid (MPA) trough level and relapse at 6 months.

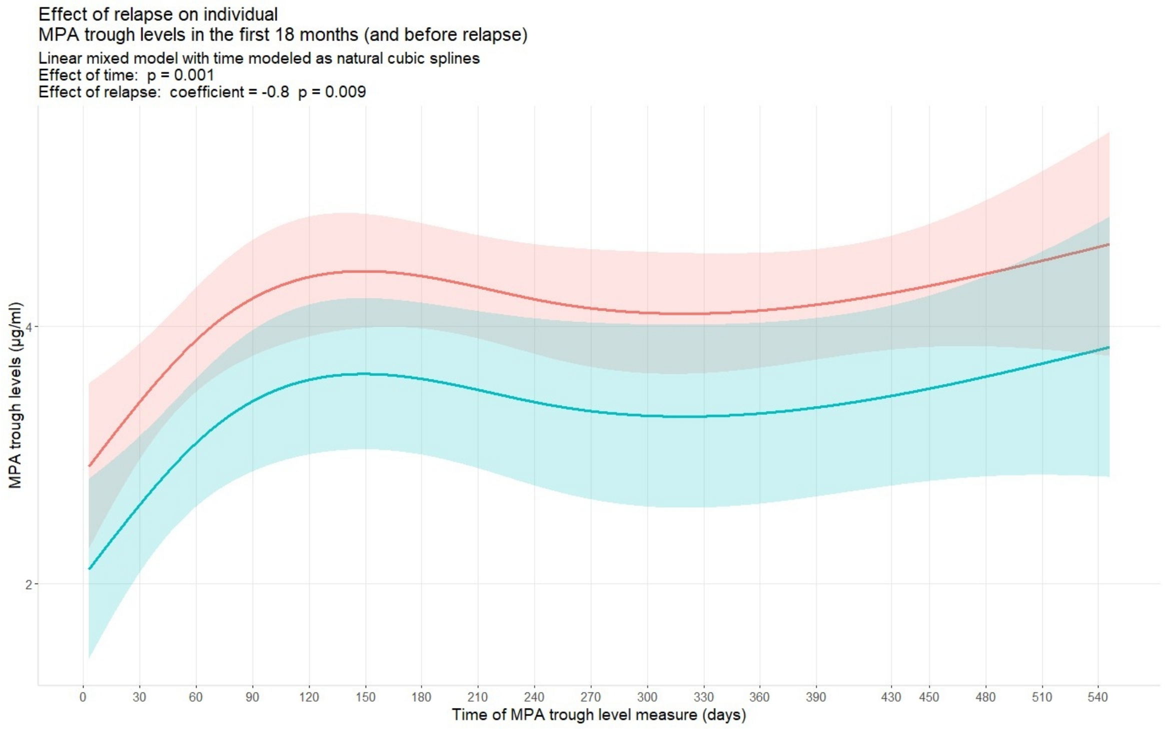

Supplementary Figure S3 - Association between mycophenolic acid (MPA) trough levels and relapse analyzed using a mixed-effects model. Individual MPA trough levels collected within the first 18 months of treatment were assessed using a mixed-effects model, accounting for repeated measures within patients. Trough levels measured after relapse events were excluded.
